# Characterization of linear accelerator X‐ray source size using a laminated beam‐spot camera

**DOI:** 10.1120/jacmp.v12i3.3463

**Published:** 2011-05-10

**Authors:** Collins Yeboah

**Affiliations:** ^1^ Department of Medical Physics Odette Cancer Centre Toronto Ontario; ^2^ Department of Radiation Oncology University of Toronto Toronto Ontario

**Keywords:** beam‐spot size, beam‐spot camera, beam‐spot stability, X‐ray source size

## Abstract

A laminated beam‐spot camera of length 20 cm and effective cross‐sectional area 2.5 cm×3 cm was designed and constructed for the measurement of X‐ray beam‐spot sizes on different models of Siemens accelerators. With the accelerator gantry at 180° and camera positioned on an accessory tray holder, an XV film placed in contact with the camera at the distal end of it detected those X‐rays that were transmitted through the camera. The FWHM of the detected X‐ray intensity profile in the gun–target (G–T) direction or the orthogonal A–B direction was used as a measure of the beam‐spot size in that direction. Siemens Mevatron MXEs exhibited a beam‐spot size of 1.7± 0.2 mm in both the in‐plane and cross‐plane directions for 6 MV photon beams. The beam‐spot size observed for a Mevatron MDX‐2 was larger by up to 1 mm, and also was different for the in‐plane and cross‐plane directions. For Siemens PRIMUS accelerators, the beam‐spot size in the in‐plane direction was found to fall in the range 2.0−2.2±0.2 mm, whereas the beam‐spot size in the cross‐plane direction fell within 1.7−1.9±0.2 mm for 6, 10, and 18 MV photon beams. Assessment of long‐term stability of the beam‐spot size shows the spot size remains fairly stable over time.

PACS number: 87.56.B‐, 87.56.bd

## I. INTRODUCTION

Megavoltage cone‐beam computed tomography (MV‐CBCT) is a volumetric X‐ray imaging modality that is being implemented as an alternative to kilovoltage cone‐beam computed tomography (kV‐CBCT) for image‐guided radiation therapy application.^(^ ^1^ ^–^ ^6^ ^)^ Unlike kV‐CBCT systems that employ a diagnostic X‐ray tube as the source of radiation, MV‐CBCT units utilize the megavoltage treatment beam from a linear accelerator for imaging. Consequently, the spatial resolution, and hence quality, of reconstructed MV‐CBCT images will be influenced, among other factors, by the size of the MV X‐ray beam‐spot; the smaller the size of the X‐ray beam‐spot, the higher the spatial resolution of the resulting volumetric image, and vice versa. Thus, gaining an insight into the magnitude and shape of the MV X‐ray beam‐spot, as well as its long‐term stability, is vital since that information will aid in the design of robust MV‐CBCT systems.

A number of studies to characterize the beam‐spot size of linear accelerators have previously been conducted.^(^ ^7^ ^–^ ^11^ ^)^ Lutz et al.^(7)^ used a laminated beam‐spot camera to characterize the beam‐spot size of an 8 MV X‐ray beam. Munro et al.^(8)^ and Jaffray et al.^(9)^ employed a CT reconstruction technique in conjunction with measured transmission through a slit at various lateral and angular positions to characterize the beam‐spot. Loewenthal et al.^(10)^ measured the beam‐spot size of Varian Clinac 1800 by using a single slit to acquire a series of slit images at various lateral positions from the beam axis, and then used these slit images and a Gaussian model of the source to estimate the source size. These studies however were performed using old generation treatment units which have since evolved to incorporate new accelerator technology. Thus, the results of the previous studies may not be applicable to the current generation of accelerators. The goal of this work was to characterize the beam‐spot size of current medical accelerators.

## II. MATERIALS AND METHODS

### A. Laminated beam‐spot camera

**Figure 1:**
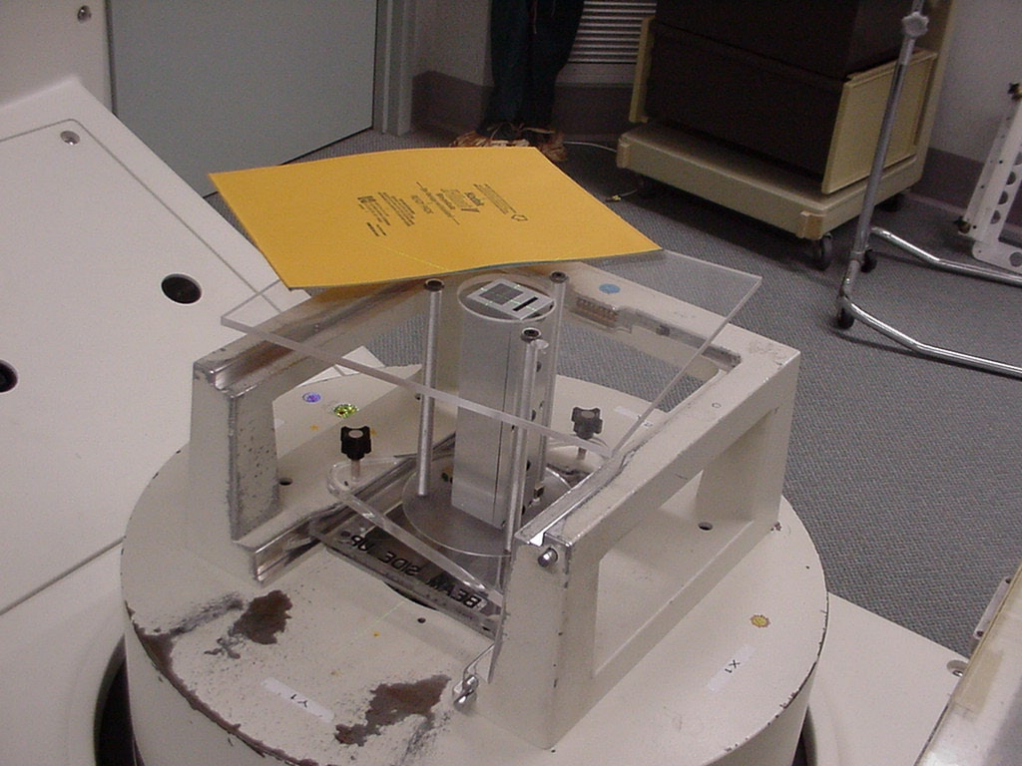
The experimental setup showing a picture of the laminated beam‐spot camera.

A laminated beam‐spot camera of length 20 cm was constructed for the measurement of the beam‐spot size. A picture of the camera is shown in Fig. 1. It consists of alternating sheets of lead and Mylar of thicknesses 0.15 mm and 0.12 mm, respectively, and of surface area 3 cm×20 cm per side. A large number of these sheets are stacked together in a machined aluminum box of length 20 cm, opened at both ends, to create a 20 cm long camera with an effective cross‐sectional area of 3 cm×2.5 cm. At the two ends of the camera where the lead and Mylar sheets are visible, the entire surfaces are machined. The principle behind the operation of such a camera has previously been described by Lutz et al.^(7)^ It makes use of the differential attenuation of X‐rays in lead and Mylar to image the X‐ray beam‐spot. Each lead sheet serves as a beam mini‐block, whereas the Mylar sheets act as “slits” through which X‐rays could freely pass. Consequently, upon incident on the camera, only those X‐rays that emerged from the X‐ray target and traveled in the direction parallel to the plane of the Mylar sheets could pass through the camera and be detected at the distal end of it; all other X‐rays will be blocked by the camera since they intercept several lead mini‐blocks. Assuming the orientation of the camera is such that the plane of the Mylar sheets is parallel to the direction of original electron pencil beam that generated the X‐rays, then the FWHM of detected X‐ray intensity profile can be taken as a measure of the beam‐spot size in the direction perpendicular to the plane of the Mylar sheets.

### B. Experimental setup and measurements

The experimental setup used to measure the beam‐spot size is shown in Fig. 1. Using a gantry angle of 180° and a field size of $4\times 4\;{\text{cm}}^{2}$, the camera was positioned in a jig that sits in the compensator tray holder and leveled, and an XV film was placed in contact with the camera on the top surface of the camera‐jig assembly. First, the camera was oriented such that the plane of the Mylar “slits” was parallel to the gun–target direction and exposures of 100 MU and 300 MU were given to two XV films, respectively. Second, the camera was rotated by 90° such that the plane of the Mylar “slits” was parallel to the A–B direction and two new XV films were exposed in sequence using 100 and 300 MU, respectively. The exposed films were developed using a Kodak X‐OMAT 2000 processor (Eastman Kodak Company, Rochester, NY) and scanned with a VIDAR VXR‐12 scanner (VIDAR Systems/Contex Group, Stockholm, Sweden) using a resolution of 89 μm. The film analysis, performed with RIT113 (Radiological Imaging Technology, Inc. Colorado Springs, CO), involved optical density‐to‐dose calibration, background subtraction, and beam‐spot intensity profile generation. FWHM of plotted beam intensity profiles in the G–T and A–B directions were used to quantify the beam‐spot sizes in these two orthogonal directions. Overall, seven accelerators were employed for this study: three Siemens Mevatron and four Siemens PRIMUS accelerators (Siemens Medical Solutions, Malvern, PA). In the remainder of this article, the terms gun–target direction and in‐plane direction are interchangeable, while the terms A–B direction and cross‐plane direction have the same meaning.

## III. RESULTS & DISCUSSION

**Figure 2:**
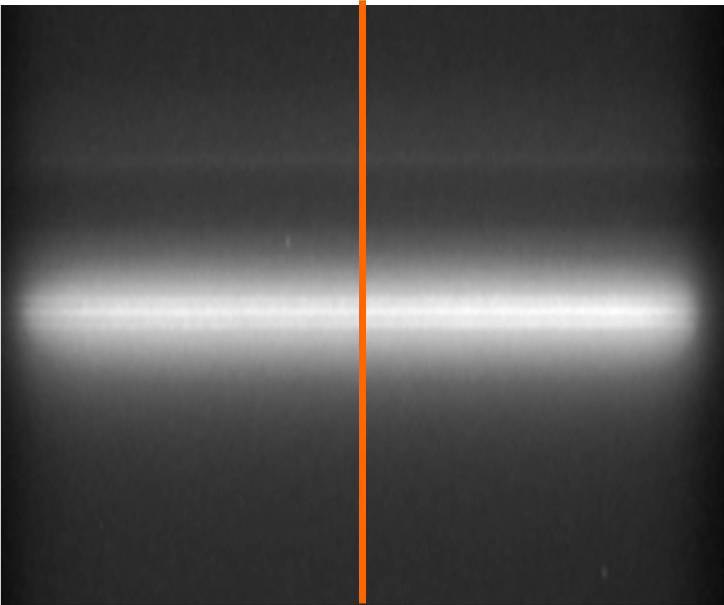
One‐dimensional image of a PRIMUS accelerator beam‐spot acquired using the laminated beam‐spot camera. The spot size in the G–T direction was derived from the relative intensity profile along the drawn red line.

**Figure 3:**
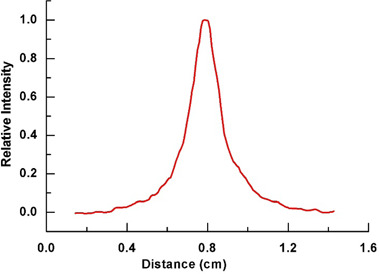
Relative intensity profile of X‐rays detected by the beam‐spot camera in the gun–target direction.

Figure 2 shows an image of the transmitted X‐ray beam through the camera when it was positioned to capture the beam intensity distribution in the in‐plane direction. The corresponding in‐plane profile of the transmitted beam is depicted in Fig. 3. The FWHM of this profile is used as a measure of the beam‐spot size in the in‐plane direction. In Table 1, the observed beam‐spot sizes (FWHM) for a number of linear accelerators and beam qualities are presented. For all the PRIMUS units investigated, the beam‐spot size was observed to fall in the range 1.7–2.2 mm for 6, 10, and 18 MV photon beams. The cross‐plane spot size fell within 1.7–1.9 mm, whereas the in‐plane spot size fell within 2.0–2.2 mm. The significance of the differences can be inferred from an analysis of the sources of experimental uncertainties, which comprise the use of finite thickness of a lead sheet (0.15 mm), resolution of the film scanner (0.089 mm), film calibration (0.1 mm), and setup error (0.1 mm). By combining these components, the overall uncertainty in the measured beam‐spot size is estimated to be 0.2 mm. Therefore, within experimental uncertainty, the measured beam‐spot size in each direction for the PRIMUS accelerators is independent of treatment unit and beam quality.

**Table 1:** Beam‐spot size measured for a number of Siemens linear accelerators in the gun–target (G–T) direction and the orthogonal A–B direction.

	*6 MV*		*FWHM (mm) 10 MV*		*18 MV*	
*Accelerator*	*G–T*	*A–B*	*G–T*	*A–B*	*G–T*	*A–B*
1. Mevatron MXE	1.7	1.8	‐	‐	‐	‐
2. Mevatron MXE	1.7	1.6	‐	‐	‐	‐
3. Mevatron MDX‐2	2.7	2.4	‐	‐	‐	‐
4. PRIMUS	2.2	1.7	‐	‐	2.0	1.9
5. PRIMUS	2.0	1.9	‐	‐	2.1	1.9
6. PRIMUS	2.0	1.8	1.9	1.8	‐	‐
7. PRIMUS	2.0	1.7	1.9	1.9	‐	‐

Also shown in Table 1 are the results for the Mevatron units, for which the measured beam‐spot sizes in the in‐plane and cross‐plane directions varied from 1.6 to 2.7 mm for 6 MV photon beams. The Mevatron MXEs presented a beam‐spot size of 1.7±0.2 mm in both the in‐plane and cross‐plane directions. However, the beam‐spot size observed for the Mevatron MDX‐2 unit was larger by up to 1 mm and also was different for the in‐plane and cross‐plane directions. Comparison of measurements performed on the Mevatron and PRIMUS units showed the beam‐spot sizes of the newer models of accelerators are not necessarily finer. In fact, of all the accelerators studied in this work, the smallest spot size in both orthogonal directions was found on a Mevatron MXE unit. Also, the spot sizes stayed fairly stable over time; measurements taken over a period of three years showed no significant differences. However, the observed spot sizes of the MV beams from Siemens accelerators are large compared to focal spot sizes of diagnostic X‐ray imaging systems; this could compromise MV‐CBCT image quality. To increase the spatial resolution of MV‐CBCT images and hence improve image quality, the beam‐spot size of Siemens PRIMUS accelerators intended for MV‐CBCT application needs to be sharpened.

This study has a number of limitations. First, the observed FWHM may include broadening of the beam‐spot that might have occurred in the camera itself.^(7)^ This effect, not accounted for in the current investigation, probably originates from the Mylar “slits” having non‐infinitesimal thicknesses and its influence may be of the same order of magnitude as the thickness of a Mylar sheet. Second, since the camera was positioned at a distance of approximately 42 cm from the photon beam source, the detected beam‐spot and the associated FWHM might include a distance‐dependent broadening effect. As explained previously by Lutz et al.,^(7)^ the magnitude of this effect probably depends on the ratio of source–camera distance to camera height. This effect was ignored in this study.
